# Impact of sex and hypoxia on brain region-specific expression of membrane androgen receptor AR45 in rats

**DOI:** 10.3389/fendo.2024.1420144

**Published:** 2024-07-18

**Authors:** Jessica L. Bradshaw, E. Nicole Wilson, Steve Mabry, Pawan Shrestha, Jennifer J. Gardner, Rebecca L. Cunningham

**Affiliations:** ^1^ Department of Pharmaceutical Sciences, University of North Texas (UNT) System College of Pharmacy, University of North Texas Health Science Center, Fort Worth, TX, United States; ^2^ North Texas Eye Research Institute, University of North Texas Health Science Center, Fort Worth, TX, United States

**Keywords:** androgen receptor, AR45, G protein, hippocampus, entorhinal cortex, sex differences, hypoxia

## Abstract

**Background:**

Sex differences in oxidative stress-associated cognitive decline are influenced by sex hormone levels. Notably, oxidative stress-associated neuronal cell death can be exacerbated through testosterone signaling via membrane androgen receptor AR45, which is complexed with G protein G_αq_ within plasma membrane-associated lipid rafts. The objective of this study was to elucidate the impact of sex on the expression of AR45 and G_αq_ in brain regions associated with cognitive function, specifically hippocampus subregions and entorhinal cortex. Additionally, we investigated whether chronic intermittent hypoxia (CIH), an oxidative stressor with sex-specific effects, would modulate AR45 and G_αq_ expression in these brain regions.

**Methods:**

Adult male and female Sprague-Dawley rats were exposed to CIH or normoxia (room air) during their sleep phase for 14 days. We quantified AR45 and G_αq_ protein expression in various cognition-associated brain regions [dorsal hippocampal CA1, CA3, dentate gyrus (DG), and entorhinal cortex (ETC)] via western blotting. For comparisons, AR45 and G_αq_ protein expression were also assessed in brain regions outside the hippocampal-ETC circuit [thalamus (TH) and striatum (STR)].

**Results:**

The highest AR45 levels were expressed in the hippocampal CA1 and DG while the lowest expression was observed in the extrahippocampal STR. The highest G_αq_ levels were expressed in the hippocampal-associated ETC while the lowest expression was observed in the extrahippocampal TH. Females expressed higher levels of AR45 in the hippocampal DG compared to males, while no sex differences in G_αq_ expression were observed regardless of brain region assessed. Moreover, there was no effect of CIH on AR45 or G_αq_ expression in any of the brain regions examined. AR45 expression was positively correlated with G_αq_ expression in the CA1, DG, ETC, TH, and STR in a sex-dependent manner.

**Conclusion:**

Our findings reveal enrichment of AR45 and G_αq_ protein expression within the hippocampal-ETC circuit, which is vulnerable to oxidative stress and neurodegeneration during cognitive decline. Nonetheless, CIH does not modulate the expression of AR45 or G_αq_. Importantly, there are sex differences in AR45 expression and its association with G_αq_ expression in various brain regions, which may underlie sex-specific differences in cognitive and motor function-associated declines with aging.

## Introduction

Membrane androgen receptors (mARs) are localized within the cellular plasma membrane and initiate an intracellular signaling cascade upon ligand binding. Currently, there are five known mARs, which are ZIP9, OXER1, GPRC6A, TRPM8, and AR45. All of these mARs, except OXER1 (1), are expressed within the brain (2–8). Specifically, ZIP9, GPRC6A, TRPM8, and AR45 are expressed within the hippocampal CA1 brain region (2, 3, 5, 6, 9), which is a major brain region that mediates learning and memory functions (10). Of note, brain mAR structures vary widely. ZIP9 and GPRC6A are seven transmembrane proteins with intracellular C-terminal domains (11, 12). TRPM8 is a cation channel within lipid rafts, which are cholesterol-rich signaling microdomains within the plasma membrane (13, 14). AR45 is a splice variant of the full-length androgen receptor (i.e., lacks N-terminal domain) that is also localized to lipid rafts of the plasma membrane (3, 15).

Prior studies examining putative mARs in the hippocampus observed insensitivity to androgen receptor inhibitors (e.g., flutamide) and increased intracellular calcium release in response to androgen receptor activation (2, 16–20). These characteristics are consistent with mARs interacting with G-protein-coupled receptors (GPCRs), such as the G_αq_ subunit that induces phospholipase C-dependent intracellular calcium release from intracellular calcium stores (21). Indeed, ZIP9, GPRC6A, TRPM8, and AR45 are associated with GPCR-G_αq_ subunit signaling and intracellular calcium release (3, 11, 14, 20, 22). Notably, GPCRs, including G_αq_ subunit, play a significant role in regulating synaptic plasticity and cognitive function (23), especially within the hippocampus wherein it can impact learning and memory processes (e.g., long-term potentiation) (24).

Upon ligand binding, stimulated mARs can activate divergent signaling cascades that elicit various phenotypic responses, including oxidative stress and cell death (2, 19, 25). Particularly, androgen-mediated signaling can be both neurotoxic and neuroprotective, depending upon the hormone receptor signaling mechanism and oxidative stress environment (2, 25, 26). For instance, androgens are neuroprotective in low oxidative stress environments and neurotoxic in high oxidative stress environments (2, 25). Our previous studies in male and female neuronal cell lines demonstrate estrogen receptor-mediated androgen signaling is neuroprotective in environments with low oxidative stress levels, while AR45-mediated androgen signaling facilitates oxidative stress-induced neurotoxicity via direct interactions with G protein G_αq_ (2, 3, 19, 20). Upon further investigation, we found that the presence of lipid rafts was integral to AR45 protein expression in neuronal cell lines (15, 27). Importantly, prior studies have shown that hypoxia elicits detrimental effects on lipid rafts through structural alterations that decrease lipid raft fluidity and permeability (28). Although we have previously shown that hypoxia can induce oxidative stress-associated cognitive decline (29–33), the mechanism through which hypoxia mediates cognitive decline is poorly understood. It is plausible that hypoxia modulates AR45 protein expression in specific brain regions via alterations in lipid rafts. Even so, the impact of oxidative stress environments on brain mAR density and signaling is unknown.

Although there are notable sex differences in oxidative stress-associated cognitive impairments (33, 34), the mechanisms underlying these sex differences are poorly understood. Our previous rat studies utilizing chronic intermittent hypoxia (CIH) as an oxidative stressor have identified sex differences in CIH-mediated cognitive dysfunction with no impact on circulating steroid hormones (33). These prior findings suggest that receptor-mediated signaling mechanisms, rather than circulating steroid hormones, may contribute to observed sex differences in cognitive dysfunction. Since AR45 and G_αq_ contribute to androgen-induced neuronal oxidative stress and neurotoxicity (2, 3, 19, 20), it is important to discern if sex differences in AR45 and G_αq_ protein expression are present in brain regions associated with cognitive function.

Overall, there are very few studies examining brain mAR protein expression across males and females, as the vast majority of mAR expression studies were conducted primarily in male mice and rats (2, 3, 5, 6). Even so, few studies have revealed the expression of TRPM8 and AR45 within male and female hippocampal regions and neurons (2, 3, 5). Within these studies, sex was used interchangeably with no statistical comparisons to determine sex differences in mAR expression patterns. Currently, no information is available regarding the impact of sex on AR45 and G_αq_ protein expression within the brain.

Altogether, the objective of this study was to elucidate the impact of sex on the expression of AR45 and G_αq_ in brain regions associated with cognitive function, specifically within hippocampal subregions and entorhinal cortex (ETC). Our laboratory has identified AR45 protein expression within the hippocampal CA1 subregion, ETC, and substantia nigra of male rats (3). However, it is unknown if AR45 is expressed in other brain regions that are involved in learning and memory, such as the thalamus (TH), striatum (STR), and hippocampal subregions CA3 and dentate gyrus (DG). Given our previous findings demonstrate AR45 is expressed in the CA1 and ETC, we examined AR45 expression in brain regions associated with CA1 and ETC circuitry. Thus, we examined AR45 and G_αq_ expression in hippocampal CA3 and DG subregions, which have excitatory projections to the ETC and CA1 (35), as well as the TH that has a weaker direct connection with the hippocampus via the hippocampal-thalamic tract (36, 37). For comparisons, we also examined AR45 and G_αq_ protein expression in the STR, which is a brain region associated with complex cognitive tasks but is not directly connected to the hippocampus (35, 36, 38–41). Additionally, we investigated whether CIH, an oxidative stressor with sex-specific effects on hippocampal-associated cognition (33, 42), would modulate AR45 and G_αq_ expression in these brain regions.

## Methods

### Animals

All protocols were approved by the Institutional Animal Care and Use Committee of the University of North Texas Health Science Center. All experiments were performed in accordance with the National Institutes of Health’s *Guide for the Care and Use of Laboratory Animals* and the ARRIVE guidelines. All experiments were conducted using virgin young adult (aged 3–4 months) Sprague-Dawley male and female rats purchased from Charles River (Wilmington, MA). Male (n = 12) and female (n = 12) rats were housed separately upon arrival in temperature and humidity-controlled rooms under 12-h:12-h reverse light/dark cycles (lights off 07:00 h, lights on 19:00 h). Reverse lighting allowed tissue collection to be conducted during the active phase of the circadian cycle. Rats were provided standard laboratory chow and water ad libitum.

### Chronic intermittent hypoxia

This study was part of a larger study examining sex-dependent effects of chronic intermittent hypoxia (CIH) on rodent hippocampal-associated cognitive function (33). One week prior to the initiation of the CIH protocol, rats (n = 6/group) were randomly assigned to either normoxia (room air) or CIH and were placed into Oxycycler chambers (76.2 x 50.8 x 50.8 cm, BioSpherix, Lacona, NY, USA) to acclimatize the rats to the chambers under normoxic conditions. The CIH protocol consisted of intermittent oxygen reduction during the rats’ sleep phase of the circadian cycle (starting at 21:00 h) as previously described (29, 30, 32, 33, 43). Briefly, oxygen was reduced from 21% (room air) to 10% in 6-minute cycles per hour (i.e., 10 cycles/hr) over 8 hrs/day for a period of 14 days.

### Euthanasia, tissue harvest, and sample preparation

At the conclusion of the CIH protocol, rats were anesthetized with 2–3% isoflurane and euthanized via decapitation during the rats’ active phase (09:00–11:00 AM). Following euthanasia, brains were quickly removed, flash-frozen in 2-methylbutane (Millipore Sigma, Cat. No. MX0760), and stored at -80°C until further analysis.

### Sample collection and preparation

Frozen brains were thawed in chilled 1X phosphate-buffered saline (Fisher, Cat. No. BP399) and sliced into 1-mm coronal sections using a brain matrix (ASI Instruments, Cat. No. RBM-4000C). Brain nuclei within the entorhinal cortex (ETC), striatum (STR), thalamus (TH), and dorsal hippocampal CA1, CA3, and dentate gyrus (DG) were microdissected according to Paxinos and Watson’s brain atlas (44) using blunt 20-gauge needles attached to 1 ml syringes. CA1, CA3, DG, ETC, and TH nuclei were collected at -5.30 mm from Bregma while STR nuclei were collected at 0.48 to -0.40 mm from Bregma. Microdissected brain nuclei were stored at -80°C until further analysis.

Frozen brain region microdissections were thawed in RIPA lysis buffer (VWR, cat #N653) containing (per 0.5 ml): 2.5 µl Halt™ protease and phosphatase inhibitor (Thermo Scientific, Ca. No. 78442), 1 µl 0.5 M ethylenediaminetetraacetic acid (EDTA, Thermo Scientific, Ca. No. J15694.AE), and 1 µl 0.5 mM dithiothreitol (DTT, Millipore Sigma, Cat. No. 43815) and homogenized as previously described (3). Total protein concentrations in brain region homogenates were quantified using Pierce BCA Protein Assay (Thermo Scientific Cat. No. 23225). Homogenized brain region samples were denatured in Laemmli buffer (Bio-RAD, Cat. No. 161–0747) containing 10% β-mercaptoethanol (Fisher, Cat. No. BP176) and heated to 95°C for 5 minutes. Denatured brain region samples were stored at -20°C until used for western blot loading.

### Western blot analysis

Equal volumes of denatured samples containing 20–25 µg of total protein were loaded onto 4–15% polyacrylamide gels (Bio-RAD, Cat. No. 4561084) and resolved in tris-glycine running buffer (Bio-RAD, Cat. No. 1610771) at 15–25 mA for 1.5 hours at room temperature. Resolved proteins were transferred to PVDF membranes at 50 V overnight at 4°C. Membranes were blocked for 1 hour at room temperature with 5% non-fat milk in 1X tris-based saline-Tween-20 (TBST, Thermo Scientific, Cat. No. 28360). Membranes were incubated overnight at 4°C with primary antibodies probing AR45 (1:1000; R&D Systems, Cat. No. MAB5876) or G_αq_ (1:250; Santa Cruz, Cat. No. sc-518023) diluted in 1% non-fat milk/TBST solutions. For protein normalization, we used β-actin primary antibody diluted in 1% non-fat milk/TBST solution (1: 2500; GeneTex, Cat. No. GTX629630) and incubated for 1 hour at room temperature. Membranes were washed in 10 minute increments in TBST for 30 minutes prior to incubation with secondary antibody. Membranes were incubated for 1 hour at room temperature with horseradish peroxidase-conjugated horse anti-mouse IgG solution (1:2500; Cell Signaling, Cat. No. 7076S) diluted in 1% non-fat milk/TBST. Immunoreactive bands were visualized using a West Pico (Thermo Scientific, Cat. No. 34580) or West Femto (Thermo Scientific, Cat. No. 34095) enhanced chemiluminescence detection assay in a Syngene G:Box imager using GeneSys Image Acquisition software (Syngene, version 1.5.2.0). Band densitometry was quantified using Image J software (National Institutes of Health, version 1.53t). AR45 (45 kD) and G_αq_ (40 kD) were normalized to β-actin (42 kD) values, and quantification of protein expression is presented as % β-actin. Unfortunately, technical difficulties with antibodies used for western blotting of microdissected brain regions containing low biomass (e.g. hippocampal subregion DG) prevented analysis of these brain regions in some rats. Representative images of western blot membranes can be found in **Supplementary Figure S1**.

### Circulating steroid hormones

Plasma levels of testosterone, estradiol, and progesterone were previously published as part of a larger study examining sex differences in hippocampal-associated cognitive function (33). Briefly, a MILLIPLEX^®^ multi-species hormone bead panel (Sigma Millipore, Cat. No. MSHMAG-21K) quantifying testosterone, estradiol, and progesterone from steroid extracted plasma samples was used. Detailed methods for the plasma extraction process and assay can be found in the previously published study. In the larger study, vaginal smears to determine estrus cycles were not performed in female rats to ensure males and females were exposed to the same stressors during behavior analyses. We previously demonstrated that plasma estradiol levels were equivalent in female rats (Mean ± SEM: 62.88 ± 4.52 pg/ml) regardless of CIH exposure (33). To examine the impact of estrus cycle on AR45 brain expression (**Supplementary Table S1**), we separated females based on low and high progesterone levels into diestrus (progesterone < 25 ng/ml) and proestrus (progesterone > 25 ng/ml).

### Data and statistical analyses

Data and statistical analyses were conducted in PRISM software (GraphPad, version 10.1). Outliers were identified and removed using robust regression and outlier removal (ROUT) with a coefficient Q = 1%. Data distributions were tested for normality using Shapiro-Wilk test. Data determined to have non-Gaussian distributions were log transformed before applying parametric statistics. Two-Way ANOVA (sex and CIH or brain region) followed by Tukey’s multiple comparisons test was used to compare group differences unless otherwise noted. The relationship between brain AR45 protein expression and brain G_αq_ protein expression or circulating testosterone was determined using Spearman correlation analysis. The significance level was set to α = 0.05, and p ≤ 0.05 was considered significant. Values are expressed as means ± SEM.

## Results

### There is no effect of chronic intermittent hypoxia on AR45 or G_αq_ protein expression in various brain regions of male and female young adult rats

**Figure 1:**
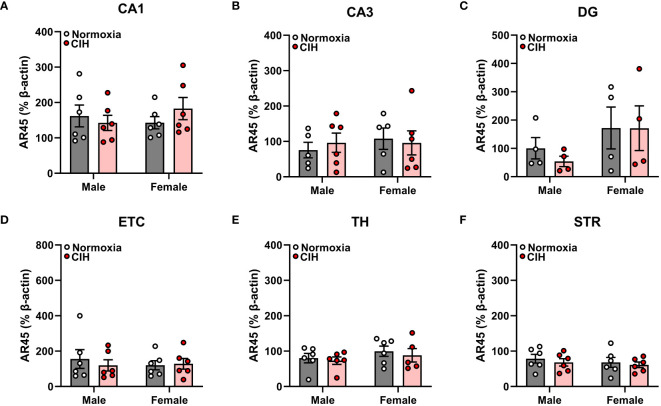
Chronic intermittent hypoxia (CIH) does not impact androgen receptor AR45 protein expression in male or female rats. AR45 protein expression was quantified as a percentage of Beta actin expression (% β-actin) in dorsal hippocampus [CA1 **(A)**, CA3 **(B)**, dentate gyrus (DG, **C**)], entorhinal cortex (ETC, **D**), thalamus (TH, **E**), and striatum (STR, **F**) brain regions from young adult male and female Sprague-Dawley rats exposed to normoxia (gray bar, white circles) or CIH (red bar and circles) for 14 days. Data were analyzed by Two-Way ANOVA (sex, CIH) with Tukey’s multiple comparisons tests; n =4–6/group.

**Figure 2:**
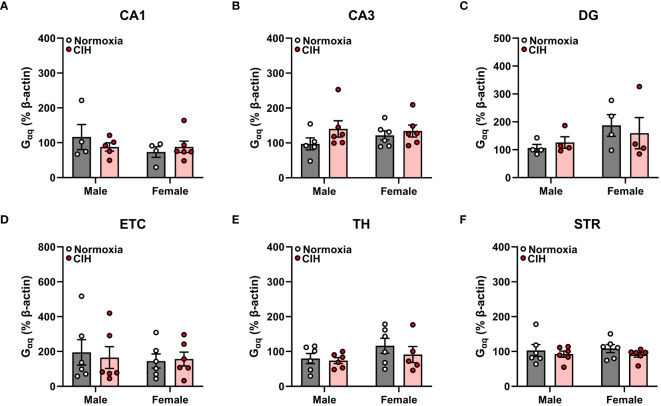
Chronic intermittent hypoxia (CIH) does not impact G_αq_ protein expression in male or female rats. G_αq_ protein expression was quantified as a percentage of Beta actin expression (% β-actin) in dorsal hippocampus [CA1 **(A)**, CA3 **(B)**, dentate gyrus (DG, **C**)], entorhinal cortex (ETC, **D**), thalamus (TH, **E**), and striatum (STR, **F**) brain regions from young adult male and female Sprague-Dawley rats exposed to normoxia (gray bar, white circles) or CIH (red bar and circles) for 14 days. Data were analyzed by Two-Way ANOVA (sex, CIH) with Tukey’s multiple comparisons tests; n =4–6/group.

**Figure 3:**
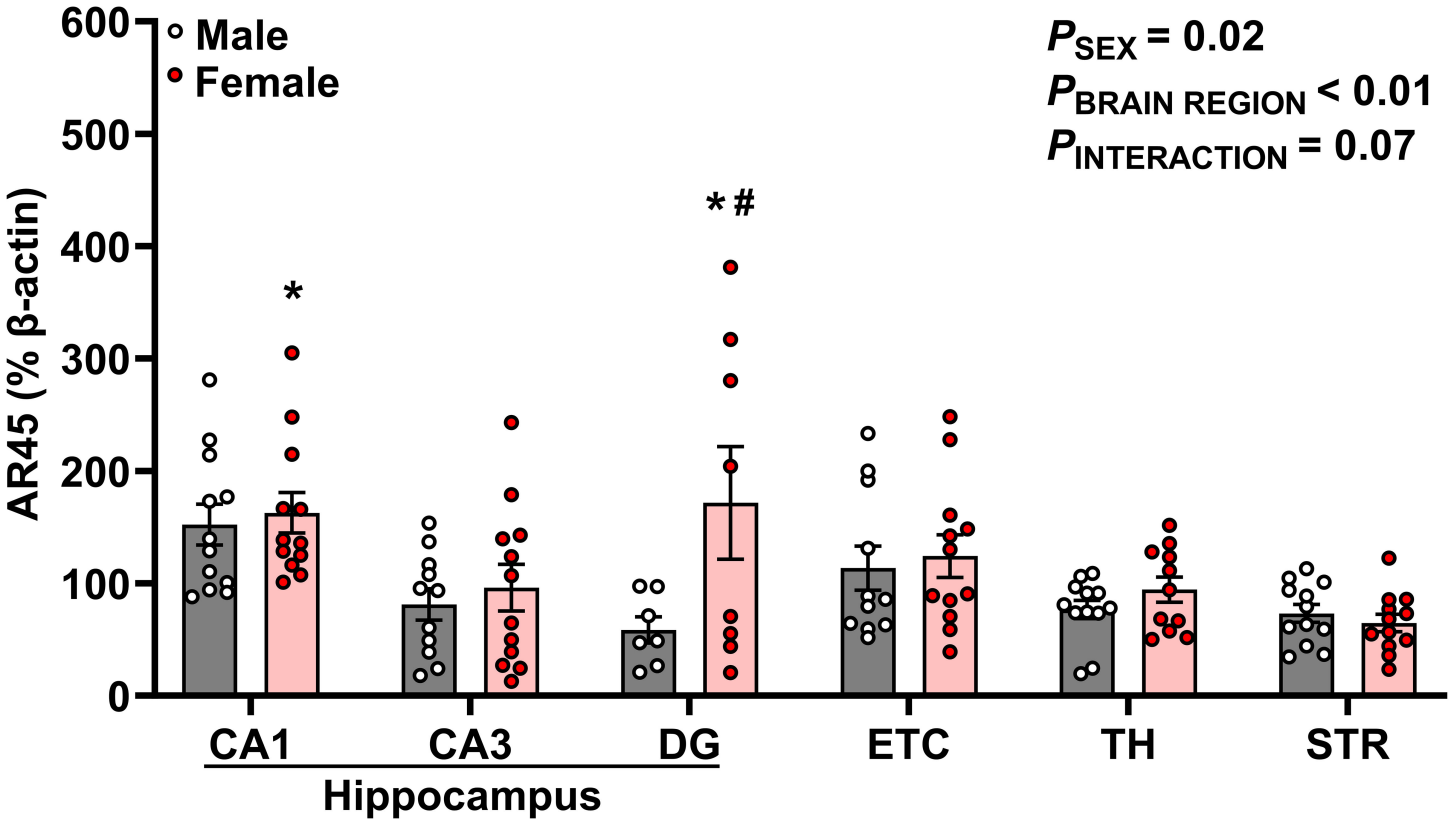
AR45 protein expression is enriched in hippocampal subregions, especially in female rats. AR45 protein expression was quantified as a percentage of Beta actin expression (% β-actin) in dorsal hippocampus [CA1, CA3, dentate gyrus (DG)], entorhinal cortex (ETC), thalamus (TH), and striatum (STR) brain regions from young adult male (gray bar, white circles) and female (red bar and circles) Sprague-Dawley rats. Data were analyzed by Two-Way ANOVA (sex, brain region) with Tukey’s multiple comparisons tests; n =7–12/group. *p < 0.05 vs. female STR; #*p* < 0.05 vs. male DG.

**Figure 4:**
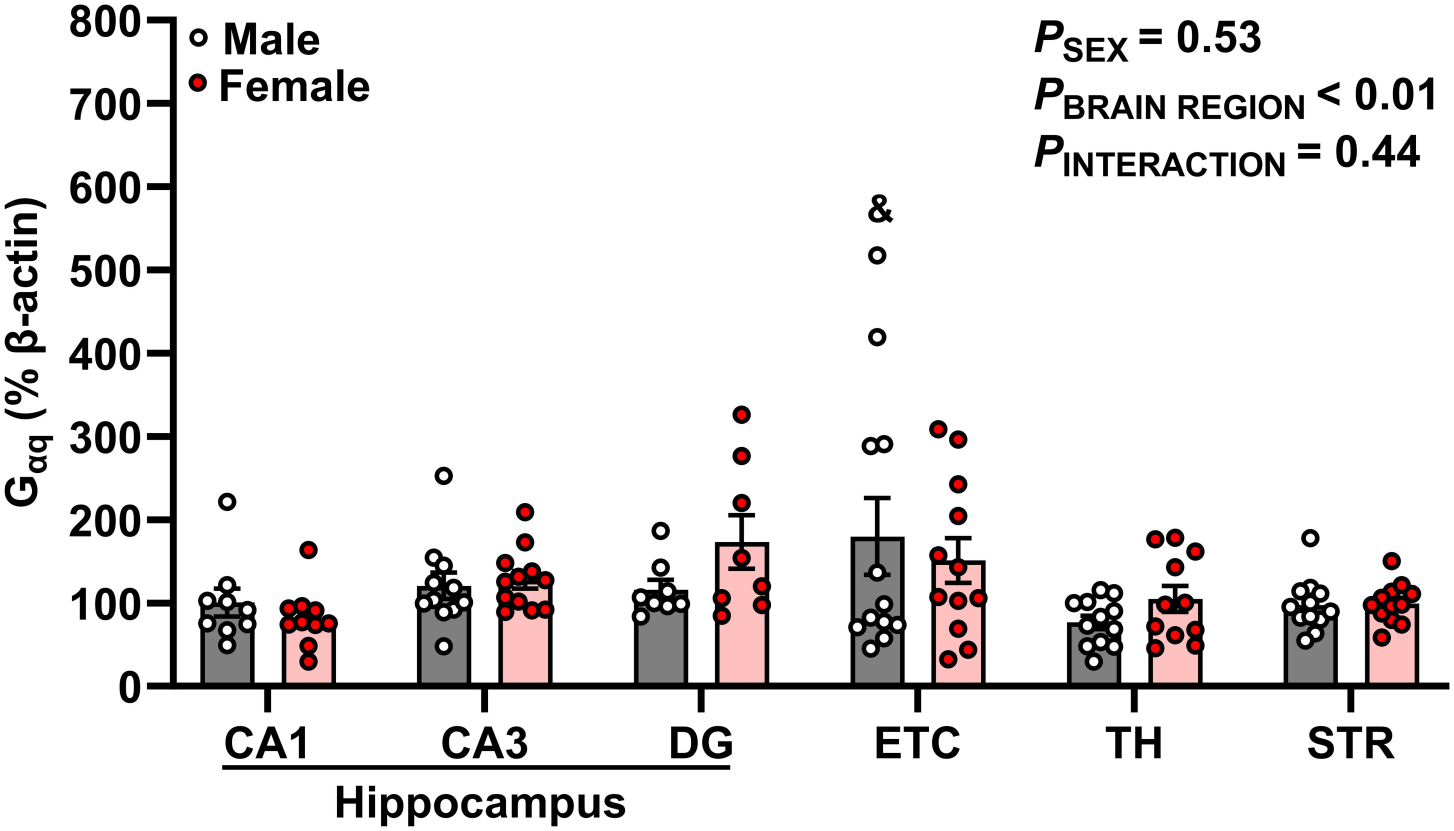
G_αq_ protein expression is enriched in hippocampal-associated entorhinal cortex. G_αq_ protein expression was quantified as a percentage of Beta actin expression (% β-actin) in dorsal hippocampus [CA1, CA3, dentate gyrus (DG)], entorhinal cortex (ETC), thalamus (TH), and striatum (STR) brain regions in young adult male (gray bar, white circles) and female (red bar and circles) Sprague-Dawley rats. Data were analyzed by Two-Way ANOVA (sex, brain region) with Tukey’s multiple comparisons tests; n = 8–12/group. &*p* < 0.05 vs. male TH.

**Figure 5:**
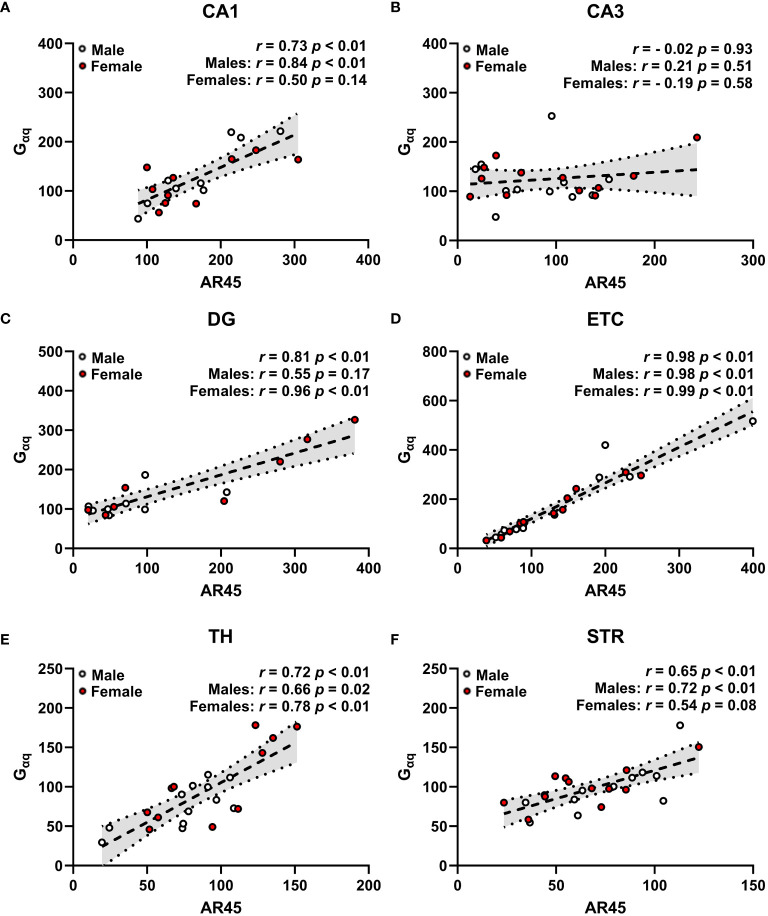
Androgen receptor AR45 and G_αq_ protein expression are correlated across brain regions in a sex-dependent manner. Scatterplots representing correlations of AR45 and G_αq_ protein expression (quantified as a percentage of Beta actin expression) in dorsal hippocampus [CA1 **(A)**, CA3 **(B)**, dentate gyrus (DG, **C**)], entorhinal cortex (ETC, **D**), thalamus (TH, **E**), and striatum (STR, **F**) brain regions from young adult male (white circles) and female (red circles) Sprague-Dawley rats. Data were analyzed by Spearman correlation with respective *r* and *p*-values listed. Dashed trend lines represent the linear relationships within the data determined by linear regression analysis. Dotted lines and shaded regions represent the 95% confidence intervals. n = 16–24/correlation plot.

We found no main effects of sex or CIH (*p* > 0.05), as well as no interactive sex and CIH effects (*p* > 0.05), on AR45 (**Figure 1**) or G_αq_ (**Figure 2**) protein expression in hippocampal regions (CA1, CA3, DG), hippocampal-associated ETC, or extrahippocampal TH or STR brain regions. Since we observed no effects of CIH on AR45 or G_αq_ expression, we collapsed normoxia and CIH groups to assess AR45 and G_αq_ expression across brain regions in male and female rats (**Figures 3**–**5**).

### AR45 protein expression is enriched in hippocampal brain regions of female young adult rats

We observed significant effects of sex (*p* = 0.015) and brain region (*p* < 0.0001) on AR45 expression (**Figure 3**). Moreover, there was a trend for interactive sex and brain region effects (*p* = 0.069). Significant brain region differences in AR45 expression were exclusively in females, with greatest expression of AR45 in hippocampal CA1 (149.8 ± 13.85%; *p* = 0.007) and DG (171.6 ± 50.15%; *p* = 0.009) compared to extrahippocampal STR (64.70 ± 7.67%). In males, there was a trend for increased AR45 expression in the CA1 (152.2 ± 18.12%) compared to DG (58.51 ± 11.79%; *p* = 0.067) and STR (73.27 ± 7.85%; *p* = 0.073). When assessing sex differences, AR45 expression was greater in female DG compared to male DG (Female: 171.6 ± 50.15% vs Male: 58.51 ± 11.79%; p = 0.022). No sex differences in AR45 expression were detected in CA1, CA3, ETC, TH, or STR (*p* > 0.05).

### G_αq_ protein expression is greatest in hippocampal-associated entorhinal cortex of male young adult rats

Our data demonstrates significant differences in G_αq_ expression across brain regions (*p* = 0.001, **Figure 4**). Specifically, G_αq_ expression was greater in male ETC compared to male TH (ETC: 179.8 ± 46.06% vs TH: 76.81 ± 8.08%; *p* = 0.028). However, we did not observe a main effect of sex or interaction between sex and brain region (*p* > 0.05).

### AR45 protein expression is positively correlated with G_αq_ protein expression across brain regions in a sex-dependent manner

Expression of AR45 positively correlated with G_αq_ protein expression in CA1 (**Figure 5A**), DG (**Figure 5C**), ETC (**Figure 5D**), TH (**Figure 5E**), and STR (**Figure 5F**), with no correlation observed in CA3 regardless of sex (**Figure 5B**). When examining sex-dependent correlations, we found that AR45 expression positively correlates with G_αq_ expression in a sex-dependent manner in hippocampal CA1 and DG (**Figures 5A, C**). Particularly, this positive association was demonstrated in male CA1 but not female CA1 (**Figure 5A**), as well as in female DG but not male DG (**Figure 5C**). AR45 expression was positively correlated with G_αq_ expression in extrahippocampal ETC and TH regardless of sex (**Figures 5D, E**), as well as in STR of males with a trend in positive association in STR of females (**Figure 5F**).

### Striatum AR45 protein expression is positively correlated with circulating testosterone levels in male rats

We next determined whether protein expression of brain AR45 was associated with circulating levels of its ligand, testosterone (**Table 1**). When examining sex-dependent correlations (male or female), we found no correlations in brain AR45 protein expression with plasma testosterone levels in female rats. Likewise, we found no correlations of circulating testosterone with AR45 protein expression in the hippocampus (CA1, CA3, DG), ETC, or TH of male rats. However, we did observe a positive correlation between STR AR45 protein expression and plasma testosterone levels in male rats. Although plasma testosterone levels were not correlated with brain AR45 protein expression in female rats, we also examined whether estrus status impacted brain AR45 protein expression in female rats (**Supplementary Table S1**). We found no effects of estrus status on AR45 brain expression within the hippocampal subregions or ETC when comparing female rats with low progesterone (diestrus) or high progesterone (proestrus).

**Table 1:** Correlations of plasma testosterone levels and brain androgen receptor AR45 protein expression across brain regions.

Brain Region	Males *n = 6–9*		Females *n = 4–9*	
	*r*	*p*	*r*	*p*
CA1	-0.333	0.385	<0.001	>0.999
CA3	0.167	0.703	0.184	0.634
DG	-0.429	0.419	-0.600	0.350
ETC	-0.067	0.880	0.586	0.183
TH	0.550	0.133	0.200	0.917
STR	0.783	0.017	-0.524	0.197

DG, dentate gyrus; ETC, entorhinal cortex; TH, thalamus; STR, striatum. Data were analyzed by Spearman correlation with respective *r* and *p*-values listed. Shaded cells represent significant correlations, *p* ≤ 0.05.

## Discussion

The major findings of this study are 1) intermittent hypoxia does not impact brain AR45 or G_αq_ expression regardless of sex, 2) AR45 protein expression was present in the hippocampus (CA1, CA3, DG), ETC, STR, and TH of male and female rats, 3) female rats express greater levels of AR45 in the DG compared to male rats, 4) no sex differences were observed in G_aq_ expression, 5) brain AR45 and G_αq_ protein expression were positively correlated in the hippocampus (CA1 and DG), ETC, STR, and TH in a sex-dependent manner, and 6) STR AR45 protein expression is positively correlated with plasma testosterone levels in male rats.

This is the first study to examine the impact of hypoxia on AR45 and G_αq_ expression. We observed no effects of intermittent hypoxia on either AR45 or G_αq_ expression in this study. It is known that hypoxia can alter the structure of lipid rafts by decreasing the fluidity and permeability of lipid rafts (28) and by increasing lipid raft-associated protein expression, such as caveolin-1 and flotillin-1, in neurons and endothelial cells (45–47). Although we found that the presence of lipid rafts is necessary for neuronal AR45 expression (15, 20), it is unclear if our intermittent hypoxia protocol is severe enough to disrupt lipid raft fluidity and permeability.

Notably, there are prominent sex differences in the prevalence and pathological onset and severity of neurological disorders (26, 34, 48, 49). Females are more likely to be diagnosed with depression, anxiety, and Alzheimer’s disease while males are more likely to be diagnosed with autism, attention-deficit/hyperactivity disorder, and Parkinson’s disease (48–51). Mounting evidence suggests sex hormones (estrogens and androgens) and associated signaling cascades contribute to emerging sex differences in the presentation and pathology of these various neurological disorders (19, 26, 52, 53). This study is the first study to examine sex differences in AR45 expression across multiple brain regions. Our previous study in male rats showed that hormone removal did not impact brain AR45 expression in CA1, ETC, or substantia nigra, but female rats were not included (3). In the present study, we found that there were no sex differences in AR45 expression in the hippocampal CA1 and CA3 regions, ETC, TH, or STR. However, we did observe sex differences in AR45 expression within the hippocampal DG, in which females expressed higher AR45 protein levels compared to males. Overall, this is the first study to observe sex differences in mAR expression, as prior studies have found no sex differences in ZIP9 (54) or TRPM8 (55) expression, and sex differences in GPRC6A expression have not been investigated.

We also observed differences in AR45 and G_αq_ expression patterns across brain regions. The highest AR45 expression was observed in the hippocampal CA1 and DG, while the lowest AR45 expression was observed in the STR. Similarly, the highest G_αq_ expression was denoted in the ETC while the lowest G_αq_ expression was denoted in the TH. Our previous work in male and female rat neuronal cell lines have demonstrated that AR45 coexpressed with G_αq_ mediates androgen’s damaging effects (2, 20). Collectively, these data suggest that AR45 and G_αq_ expression are enriched within the hippocampal-ETC circuit of male and female rats, highlighting the vulnerability of this circuit to damaging AR45-mediated testosterone signaling (2).

Our findings reveal AR45 expression is positively correlated with G_αq_ protein expression within the hippocampus (CA1 and DG), ETC, TH, and STR when examining sex-collapsed associations. When assessing correlations based on sex, we found that there are sex-dependent effects on the correlation of AR45 and G_αq_ expression across brain regions. Specifically, AR45 and G_αq_ associations within the hippocampal CA1 and STR were observed in male but not female rats. In contrast, an association between AR45 and G_αq_ was observed in the hippocampal DG of female but not male rats. These sex differences in hippocampal AR45- G_αq_ expression may be involved in the sex differences observed in CA1-mediated functions, as the CA1 has strong excitatory connections with DG and ETC (35), weaker connections with the TH (36, 37), and is not connected to the STR (35, 36, 38–41). Similar to our findings of a male-bias sex difference (AR45- G_αq_ association) in hippocampal CA1 and STR, other studies consistently observe a male-sex bias in these regions, as evidenced by increased CA1 volume (56), larger dendritic trees of CA1 pyramidal neurons (57, 58), and larger STR volume (59). In contrast, we observed female-sex bias (AR45- G_αq_ association) in the hippocampal DG and no sex differences in the ETC and TH. Previous findings of sex differences in the DG (60–63) and the ETC (64, 65) are generally male-sex biased, whereas sex differences in the TH range from larger TH volume in males (66), larger TH volume in females (67), to no sex differences in the TH volume (59). Regardless of sex, we observed no AR45- G_αq_ association in the CA3 region, which is consistent with findings showing no sex differences in CA3 volume (56).

Lastly, we identified a positive correlation between STR AR45 protein expression and circulating testosterone levels in male rats. There were no correlations between circulating testosterone levels and brain AR45 expression in hippocampal subregions, ETC, or TH of male rats. These findings build upon our previous findings demonstrating no effects of gonadal hormones on AR45 expression in hippocampal CA1, ETC, or substantia nigra of male rats (3). Previous studies have demonstrated increases in circulating testosterone levels are associated with STR-mediated impulsive and risky behaviors in male rats (68). Thus, the expression of AR45 in the STR of male rats may facilitate circulating testosterone’s effects on male decision making and social behavior. Furthermore, this is the first study to examine the impact of circulating hormones on AR45 expression in female rats. We found no correlations between brain AR45 protein expression and plasma testosterone levels in any of the brain regions examined in female rats. When we examined the impact of low and high plasma progesterone levels on brain AR45 expression in female rats with equivalent plasma estradiol levels, we also observed no effects of low or high plasma progesterone levels on brain AR45 levels in hippocampal subregions or the ETC of female rats.

### Limitations and future directions

Due to a lack of sufficient protein concentrations in brain region samples, we were unable to examine AR45-associated protein-protein interactions and downstream signaling cascades. Moreover, we did not assess AR45 and G_aq_ expression in various cell types (i.e., glial cells and neurons) within the brain regions. Still, previous studies have not identified sex differences at the level of neuronal characteristics, such as spine density in the CA1, CA3, DG (69), or neuronal density and soma size in the STR (70, 71). Although we were unable to examine full-length androgen receptor (FL-AR) due to degradation of FL-AR during sample processing (3), previous studies have shown increased expression of FL-AR in the STR, CA1, DG, and cerebral cortex in males with equivalent levels in CA3 and TH in males and females (72). Future studies could assess AR45 and G_aq_ coexpression by diverse cell types across brain regions, as well as activation of various signaling cascades and associated cellular outcomes. Additionally, we utilized a mild intermittent hypoxia protocol to investigate the effects of an oxidative stressor on AR45 expression. Thus, the impact of more stringent brain-targeting hypoxia protocols, such as sustained hypoxia or ischemic stroke, on AR45 expression and functional outcomes are warranted. Furthermore, future studies examining the impact of brain AR45 expression and stimulation on cognition and behavioral outcomes are needed to understand physiological mechanisms and identify possible avenues for intervention. Although our prior studies observed no effect of aging [young adult (3 month) vs middle-age (9–12 months)] on brain AR45 expression in male rats (3), it remains unknown how aging and reproductive status (e.g., pregnancy, menopause) affect female brain AR45 expression. Furthermore, our prior studies did not investigate brain AR45 expression prior to adulthood (3). Thus, future studies examining the impact of development and puberty on brain AR45 expression in males and females are warranted.

### Perspectives and significance

Our findings reveal AR45 and G_aq_ are differentially expressed across brain regions, suggesting that specific brain regions may be more vulnerable to the damaging effects of AR45-mediated testosterone signaling in oxidative stress environments. Moreover, females exhibited greater expression of brain AR45 compared to males within the hippocampal DG, a brain region associated with depression and the formation of memories (73–75). Thus, women with higher levels of circulating testosterone compared to protective estrogen levels, such as in hyperandrogenism conditions (e.g., polycystic ovarian syndrome) and menopause, may be more vulnerable to testosterone-mediated memory and affective behavior impairments. Moreover, our findings demonstrate male circulating testosterone levels are positively correlated with AR45 protein expression in the STR, a brain region associated with motor function, as well as social decision-making behavior and impulsivity (76, 77). Therefore, instances in which circulating testosterone increases (e.g., puberty) or decreases (e.g., aging), may elicit AR45-mediated modifications to STR-associated behaviors, such as movement and rational behaviors.

## Data availability statement

The original contributions presented in the study are included in the article/**Supplementary Material**. Further inquiries can be directed to the corresponding author.

## Ethics statement

The animal study was approved by Institutional Animal Care and Use Committee of the University of North Texas Health Science Center. The study was conducted in accordance with the local legislation and institutional requirements.

## Author contributions

JB: Conceptualization, Data curation, Formal analysis, Investigation, Methodology, Supervision, Validation, Visualization, Writing – original draft, Writing – review & editing. EW: Data curation, Formal analysis, Investigation, Methodology, Writing – review & editing. SM: Data curation, Investigation, Methodology, Writing – review & editing. PS: Data curation, Investigation, Writing – review & editing. JG: Data curation, Investigation, Writing – review & editing. RC: Conceptualization, Funding acquisition, Project administration, Resources, Supervision, Writing – original draft, Writing – review & editing.
