# Supplementary material for: Impact of sex and hypoxia on brain region-specific expression of membrane androgen receptor AR45 in rats
Source: Front Endocrinol (Lausanne). 2024 Jul 18;15:1420144. doi: 10.3389/fendo.2024.1420144 (PMC11291194; doi:10.3389/fendo.2024.1420144)
Supplement: Supplementary file 1 [file DataSheet_1.docx]

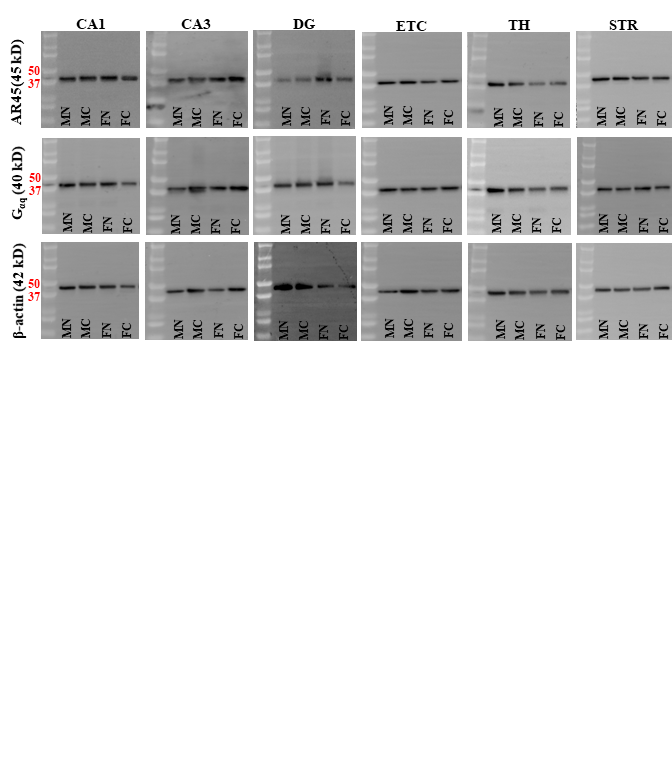
Supplementary Material

**Figure S1. Representative western blot images.** Androgen receptor AR45 and G_αq_ protein expression were probed in hippocampus [CA1, CA3, dentate gyrus (DG)], entorhinal cortex (ETC), thalamus (TH), and striatum (STR). Protein expression was normalized to percentage of β-actin expression. Standard bands indicating 50 and 37 kD are denoted in red. Lane samples are labeled according to group: male normoxia (MN), male chronic intermittent hypoxia (MC), female normoxia (FN), and female chronic intermittent hypoxia (FC). Each lane represents a single rat from its respective group (no pooling of samples was performed).

**Table S1. Plasma progesterone levels do not impact AR45 protein expression.**

|  | **AR45 protein expression (% β-actin)** | |
| --- | --- | --- |
| **Brain Region** | **Low Progesterone**  **(Diestrus)** | **High Progesterone (Proestrus)** |
| **CA1** | **174.8 ± 25.08** | **121.6 ± 7.54** |
| **CA3** | **87.83 ± 25.48** | **112.6 ± 42.69** |
| **DG** | **193.8 ± 123.2** | **99.93 ± 60.53** |
| **ETC** | **108.7 ± 19.55** | **143.1 ± 41.55** |

DG = dentate gyrus, ETC = entorhinal cortex. Data were analyzed by unpaired t-test or Mann-Whitney test (n = 2-5/group). No significant differences (*p* > 0.05) were observed. Data represents mean ± SEM. Low progesterone (< 25 ng/ml) = diestrus stage; high progesterone (> 25 ng/ml) = proestrus stage.
